# Mistaking Spontaneous Recoveries for Therapeutical Results

**Published:** 1873-06

**Authors:** 


Mistaking Spontaneous Recoveries for Therapeutical Results.—Grave errors are often committed in attributing cures to the result of treatment, and sometimes important curative means receive very little consideration. The recovery of patients affected with intermittent fever, under mercurial treatment, in vogue half a century ago, was attributed to the ptyalism, while in truth it was a decidedly injurious complication, coming on as the febrile excitement began to decline, but believed by the physician and patient to be the cause of such decline. Gastritis finds its most potent remedy in the demulcent drink to which very little importance is attached.

"The thousand and one" nostrums vended to the unsuspecting subjects of slight ailments, for which a little time and patience are sufficient, are magnified by them into the most certain curatives.

For the cure of rheumatism, ague, and other diseases which often run their course and subside spontaneously, even under embarrassing treatment, we find diversified and conflicting plans of treatment instituted. Statistics of cure are reported in connection with all of them, and full confidence expressed by each reporter.

The greater the humbug and the more unnatural and unreasonable the means of cure, the more attractive to invalids, and truth compels us reluctantly to say, even physicians themselves partake of this fancy for delusion.

The *Reporter* for May, 1873, transfers from an English exchange the tale of not only an imaginary cure, but the transfer of ague to the object of the patient's dislike. A man reduced by chills to supposed peril, bequeathed, in his will, to the parish parson, "these plaguey fits of the ague." He at once recovered, after indulging in laughter over the prospective pleasure of having this part of his will executed before death. The clergyman, highly offended at the bequest, was attacked next day with persistent chills.

Fodder tea, a grain of black pepper, and, in fact, whatever is said or done last before the chills cease, is set down as the cure, by the credulous and imaginary.

